# Genome sequencing in families with congenital limb malformations

**DOI:** 10.1007/s00439-021-02295-y

**Published:** 2021-06-22

**Authors:** Jonas Elsner, Martin A. Mensah, Manuel Holtgrewe, Jakob Hertzberg, Stefania Bigoni, Andreas Busche, Marie Coutelier, Deepthi C. de Silva, Nursel Elçioglu, Isabel Filges, Erica Gerkes, Katta M. Girisha, Luitgard Graul-Neumann, Aleksander Jamsheer, Peter Krawitz, Ingo Kurth, Susanne Markus, Andre Megarbane, André Reis, Miriam S. Reuter, Daniel Svoboda, Christopher Teller, Beyhan Tuysuz, Seval Türkmen, Meredith Wilson, Rixa Woitschach, Inga Vater, Almuth Caliebe, Wiebke Hülsemann, Denise Horn, Stefan Mundlos, Malte Spielmann

**Affiliations:** 1 Institute of Medical Genetics and Human Genetics, Charité-Universitätsmedizin Berlin, Corporate member of Freie Universität Berlin and Humboldt-Universität zu Berlin, Berlin, Germany; 2 Berlin Institute of Health at Charité – Universitätsmedizin Berlin, Berlin, Germany; 3 Core Unit Bioinformatics, Berlin Institute of Health (BIH), Berlin, Germany; 4 Max Planck Institute for Molecular Genetics, RG Development and Disease, Berlin, Germany; 5 Medical Genetics Unit, Department of Mother and Child, Ferrara Sant’Anna University Hospital, Ferrara, Italy; 6 Institut Für Humangenetik, Universitätsklinikum Münster, Münster, Germany; 7 Department of Human Genetics, Faculty of Medicine, Jewish General Hospital, McGill University, Montreal, QC Canada; 8 Faculty of Medicine, University of Kelaniya, Ragama, Sri Lanka; 9 Department of Pediatric Genetics, Marmara University Medical School, Istanbul, Turkey; 10 Eastern Mediterranean University Medical School, Cyprus, Mersin 10, Turkey; 11 Institut für Medizinische Genetik und Pathologie, Universitätsspital Basel, Basel, Switzerland; 12 Department of Genetics, University of Groningen, University Medical Center Groningen, Groningen, The Netherlands; 13 Department of Medical Genetics, Kasturba Medical College, Manipal Academy of Higher Education, Manipal, India; 14 Department of Medical Genetics, Poznan University of Medical Sciences, Poznan, Poland; 15 Institute for Genomic Statistics and Bioinformatics, University of Bonn, Bonn, Germany; 16 Institute of Human Genetics, Medical Faculty, RWTH Aachen University Hospital, Aachen, Germany; 17 Fachärztin Für Humangenetik, Bischof-von-Henle-Straße 2a, Regensburg, Germany; 18 Department of Human Genetics, Gilbert and Rose-Marie Chagoury School of Medicine, Lebanese American University, Byblos, Lebanon; 19 Institute of Human Genetics, Friedrich-Alexander-Universität Erlangen-Nürnberg, Erlangen, Germany; 20 Kinderhandchirurgie, Medizinische Fakultät Mannheim der Universität Heidelberg, Heidelberg, Germany; 21 Synlab MVZ Bad Nauheim, Mondorfstr. 1761231, Bad Nauheim, Germany; 22 Department of Pediatric Genetics, Cerrahpasa Medical Faculty, Istanbul University-Cerrahpasa, Istanbul, Turkey; 23 National Center of Genetics (NCG), Laboratoire National de Santé 1, Rue Louis Rech, 3555 Dudelange, Luxembourg; 24 Genetic Medicine, Children’s Hospital at Westmead, Paediatrics and Child Health, Sydney, Australia; 25 Institute of Human Genetics, University Medical Center Hamburg, Eppendorf, Germany; 26 Institute of Human Genetics, University of Kiel, Kiel, Germany; 27 Katholisches Kinderkrankenhaus Wilhelmstift, Hamburg, Germany; 28 Institute of Human Genetics, University of Lübeck, Lübeck, Germany

## Abstract

The extensive clinical and genetic heterogeneity of congenital limb malformation calls for comprehensive genome-wide analysis of genetic variation. Genome sequencing (GS) has the potential to identify all genetic variants. Here we aim to determine the diagnostic potential of GS as a comprehensive one-test-for-all strategy in a cohort of undiagnosed patients with congenital limb malformations. We collected 69 cases (64 trios, 1 duo, 5 singletons) with congenital limb malformations with no molecular diagnosis after standard clinical genetic testing and performed genome sequencing. We also developed a framework to identify potential noncoding pathogenic variants. We identified likely pathogenic/disease-associated variants in 12 cases (17.4%) including four in known disease genes, and one repeat expansion in *HOXD13*. In three unrelated cases with ectrodactyly, we identified likely pathogenic variants in *UBA2*, establishing it as a novel disease gene. In addition, we found two complex structural variants (3%). We also identified likely causative variants in three novel high confidence candidate genes. We were not able to identify any noncoding variants. GS is a powerful strategy to identify all types of genomic variants associated with congenital limb malformation, including repeat expansions and complex structural variants missed by standard diagnostic approaches. In this cohort, no causative noncoding SNVs could be identified.

**Supplementary Information:**

The online version contains supplementary material available at 10.1007/s00439-021-02295-y.

## Introduction

The repertoire of diagnostic tests in human genetics is as diverse as the types of genetic alterations they were developed to detect (Berisha et al. 2020). Through the development of Next Generation Sequencing technologies (NGS) sequencing has become several orders of magnitude faster and cheaper. This has led to an enormous increase in the efficiency of genetic testing (Levy and Myers 2016). NGS quickly found its way from research applications to the clinic: today, panel and exome sequencing are elements of the routine diagnostics in genetic medicine (Deciphering Developmental Disorders Study 2017). Despite these significant advances, classical genetic testing methods such as chromosomal microarray analysis (CMA) and Sanger sequencing remain part of the standard diagnostic arsenal. This is because NGS-based gene panels often do not detect structural variants such as inversions and translocations, or fail to determine repeat lengths (Berisha et al. 2020). The goal of detecting all types of genetic variation in a single test can theoretically be achieved by short-read based genome sequencing (GS) (Xue et al. 2015). While there are some very encouraging proof of concept studies for the use of GS in individuals with intellectual disability (Lindstrand et al. 2019), GS is not yet part of the clinical routine and there is a lack of systematic studies on the benefits of such tests for individuals with congenital malformations.

A major limitation of panel and exome sequencing approaches is that they usually do not cover 98% of the genome which is noncoding, and are, hence, unable to detect deep intronic splice variants or intergenic regulatory variants. Therefore, over 40% of individuals with genetic diseases receive no molecular diagnosis after standard testing (Gilissen et al. 2014). This is likely because the noncoding sequence has largely been ignored despite most nucleotides and single nucleotide variants being noncoding. The two main challenges that currently hamper the medical interpretation of noncoding variants are the poor understanding of the “regulatory code” of the noncoding genome and the large number of noncoding variants in each individual that renders classical functional work-up strategies impossible.

In this study, we aimed to determine the diagnostic potential of GS as a comprehensive one-test-for-all strategy in a cohort of 69 unsolved patients with congenital limb malformations. We also attempted to develop a framework to prioritize the large number of noncoding variants identified in the GS studies by combining mouse genetic and human functional epigenetic data with in vivo-validated enhancer sequences.

## Materials and methods

### Study design

Patients affected with malformations of two limbs, or two individuals from a family, each affected with a malformation of at least one limb were recruited (Supplementary Fig. 1). Exclusion criteria included a molecularly established genetic diagnosis, a suspected diagnosis of amniotic band syndrome, or an isolated fifth finger clinodactyly. A convenience set of samples was collected from the patients of the Department of Hand Surgery of the Katholisches Kinderkrankenhaus Wilhelmstift Hamburg and the Institute of Medical Genetics and Human Genetics of the Charité (IMG)—Universitätsmedizin Berlin. This sample-set was compiled with cases that were sent to the IMG by external physicians for diagnostic purposes. The sample-set was fixed before conducting GS.

### Included patients

We included 69 patients in this study (Supplementary Table 1). We sequenced the index case and both parents in 64 cases, the index and one parent in one case, and only the index in four cases (parental DNA was not available for testing). In one case, we additionally sequenced a sibling. In five cases, one parent showed a limb malformation comparable to the index. In one case featuring ectrodactyly and apparently unaffected parents and grandparents, a maternal grand-uncle was affected, who was also sequenced. In 60 cases no family member other than the index was reported to show a limb malformation.

### Phenotyping and conventional genetic testing

Limb malformations were phenotyped based on photographs and radiographs by a panel of medical professionals including expert clinical geneticists. Phenotypes were described as per Human Phenotype Ontology (HPO) terminology.

Based on a patient's phenotype, genes were selected for sequencing by medical geneticists. Sample preparation and Sanger-sequencing were performed using standard procedures. High resolution (1 M oligo) CMA was performed as described previously (Flöttmann et al. 2018).

### Genome sequencing and variant calling

Paired-end PCR-free GS was performed by Macrogen Inc. (South-Korea) using a HiSeq X Ten platform. DNA preparation, sequencing, and sequence data processing were performed according to Macrogen’s standard protocol (coverage 30× and read length 150 bp).

The FASTQ files were transferred to the Core Unit Bioinformatics of the Berlin Institute of Health (CUBI) for variant calling. Files were further processed and securely stored in the System for Omics Data Analysis and Retrieval (SODAR) (Nieminen et al. 2020). GATK HC was used to call simple nucleotide variants, while structural variants were called using Delly2, PopDel, and ERDS/SV2. Afterwards, variants were processed and annotated by the VarFish platform (Holtgrewe et al. 2020). Variants were mapped according to the hg19 reference genome.

### Variant filtration

#### Coding SNVs and SVs

Each index case was filtered as a singleton, regardless of the availability of family data. If parental samples were available, a trio-based filtration approach was additionally performed. Male-index trio-cases were also filtered for hemizygous X-chromosomal variants.

Simple nucleotide variant filtration was performed on the VarFish platform (Version v0.17.2) (Holtgrewe et al. 2020). We filtered GS data for non-synonymous exonic and splice variants using default settings for read depth, allelic balance, and read quality. Allele counts were set as described in Supplementary Table 2. SNVs that passed filtration were exported as variant calling files (VCF). For evaluation of variant pathogenicity, VCFs were uploaded to MutationDistiller and Exomiser. The first ten results were exported for semi-automated, in-depth analysis (see Supplementary Fig. 2 for details). We also tested for truncating or probable LoF (CADD > 20) variants affecting the same gene with a pLI > 0.9 in at least two independent patients.

Structural variant filtration was performed as described in Supplementary Table 3. The minimal size for structural variant filtration was 1500 bp. Whenever we obtained more than 30 structural variants after initial filtering, we increased the number of minimally covered informative reads from 2 to 5. Each SV passing filtration was judged manually with the information provided by the IGV-Browser and UCSC (see Supplementary Fig. 3 for details).

All findings were evaluated at weekly clinical meetings.

#### Analysis of noncoding variants: limb regulome

We defined a limb-specific potential regulome to filter and interpret non-coding variants. For this purpose, we created a list of 1719 genes involved in embryonic limb development based on data from the Mouse Genome Informatics (MGI) database and entries in OMIM. We defined the human limb regulome as the following: 1. all conserved (phyloP > 1,3) variants, 2. those located within the same topologically associating domain (TAD) (as determined in human fibroblasts (Dixon et al. 2012)) as a limb gene, 3. those that were marked by an H3K27 acetylation peak in human limb buds (Cotney et al. 2013). We also included the validated enhancer elements of the VISTA Enhancer Browser. Coding and noncoding SNVs were filtered for rare variants (frequency < 0.1%) and were considered to be potentially affecting the same regulatory element if they were either less than 300 bp apart or positioned in the same established enhancer.

Whenever we identified rare, potentially pathogenic heterozygous coding variants of genes associated with a recessive limb phenotype in individuals featuring at least a partial overlap with that phenotype, we also screened for *in trans* conserved non-coding variants with a MAF < 3% affecting the same TAD.

## Results

We collected a cohort of 69 individuals affected with congenital limb malformations. All individuals had previously gone through our clinical genetics routine pipeline including clinical examination, candidate gene testing, and CMA. We then performed GS as a comprehensive one-test-for-all strategy.

In total, we identified 333,163,643 single nucleotide variants (SNVs) among the 69 sequenced index patients, of which 7,020,766 were either coding or flanking coding elements by 10 bp or less. 326,142,877 were noncoding SNVs, of which 19,362 were rare (gnomAD AF < 0.01). 21,369 of the coding SNV calls were classified by Jannovar to be of at least moderate relevance (missense and truncating), and 1429 to be of high relevance (truncating only). VarFish filtering returned 5761 SNVs. Filtering by Exomizer and MutationTaster identified 433 potentially pathogenic coding calls among these, of which 174 were high-quality calls suitable for further evaluation.

30,062, SNVs resulted in the potential loss-of-function of probably haploinsufficient genes. 49 of these affected the same gene in two unrelated index individuals (Supplementary Fig. 2).

We also analyzed the structural variants in 68 of the 69 index patients. 55 cases were filtered as trios with unaffected parents and moderate filter settings. Five were filtered as trios with another affected relative and moderate filter settings. Stricter filter settings were chosen for 9 trios because moderate filter settings produced an unmanageable amount of SV calls. 3 cases were analyzed as singletons. Individuals I1, I2, I3 did not yield any results, I4 was excluded from the SV analysis because too many SVs were called even with stricter filter settings due to poor data quality.

Of the 1,555,426 SVs, 633 SVs passed the filtering by VarFish, of which 222 were inversions, 288 deletions, 76 duplications, and 47 breakpoints of potential translocations.

417 of these SVs were excluded because they were of poor calling quality or because they were inherited from an unaffected parent. We then manually inspected the remaining 216 SVs. Segregation analysis in the parents was performed by qPCR after comparing candidate CNVs with known limb genes according to the Human Phenotype Ontology, cross-species phenotype comparison, mouse models, gene expression data (Cao et al. 2019), limb enhancer elements (Visel et al. 2009), and the local topological associating domain (TAD) architecture of the locus (Dixon et al. 2012; Cao et al. 2019). As a result, we identified 30 promising variants (Supplementary Fig. 3).

### Variants in four known, limb malformation associated genes

We identified pathogenic variants in established disease genes in four individuals (I5, I6, I7, I8, Supplementary Fig. 4), which we confirmed by Sanger sequencing. These included a missense variant in *FGFR1,* already described in the literature (Muenke et al. 2014), and three previously undescribed variants in the genes *FGFR2*, *GLI3*, and *BHLHA9*. In all four cases, we classified the variants as (likely) pathogenic according to the criteria of the American College of Medical Genetics and Genomics (ACMG), based on the type of variant and the phenotype of the patient. All variants were inherited (note that the mother of I8, was not radiographically phenotyped, which is necessary to diagnose mild *FGFR2*-associated phenotypes (Flöttmann et al. 2015)).

#### Repeat expansions of *HOXD13*

In individual I9, featuring brachy-poly-syndactyly, we detected a repeat expansion by eight alanines on the *HOXD13* allele inherited from his affected mother, already described as pathogenic in the literature (Brison et al. 2014), and a polymorphic repeat expansion by only one alanine on the paternal *HOXD13* allele (Supplementary Fig. 5). These findings were confirmed by conventional *HOXD13* microsatellite analysis.

#### Structural variants at known disease loci

**Fig. 1:**
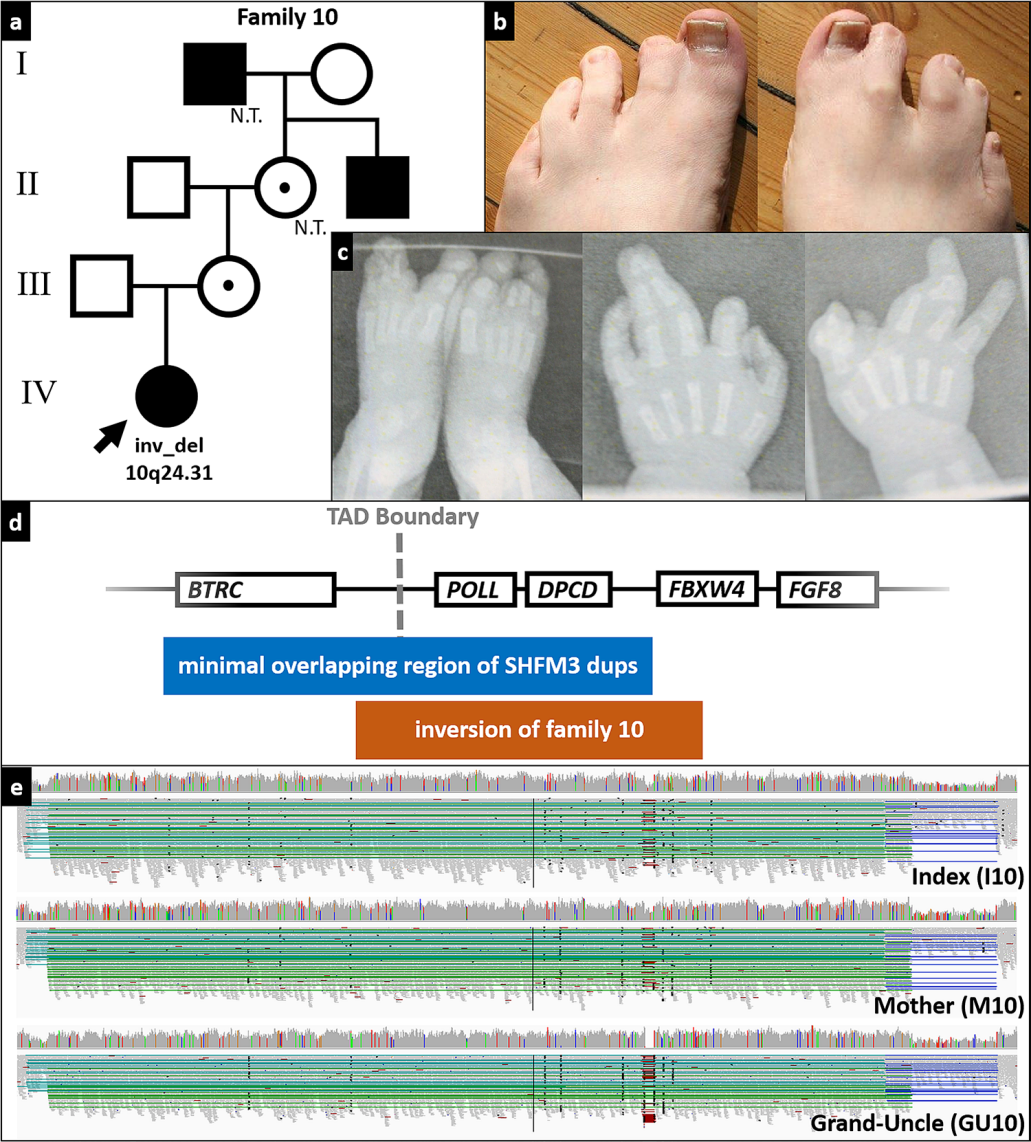
Inversion-deletion at SHFM3 locus: **a** pedigree, N.T. not tested. **b** feet of grand-uncle (II-3). **c** hands and feet of the index patient (IV-1). **d** genomic architecture of SHFM3. **e** GS data of the family, note the presence of an inversion (chr10: 103,321,526–103,426,609) flanked by deletions (chr10:103,319,219–103,321,525 and chr10:103,426,610–103,436,718) on either site

In individual I10 with bilateral upper and lower limb ectrodactyly, we identified an inversion of 105 kb (chr10: 103,321,526–103,426,609) flanked by two deletions (chr10:103,319,219–103,321,525 and chr10:103,426,610–103,436,718) at the split-hand foot malformation locus 3 (SHFM3) on chr10q24 inherited from his unaffected mother (Fig. 1, Supplementary Fig. 6). His affected great-uncle also carried the inversion. The variant overlaps with the most common duplications associated with ectrodactyly (de Mollerat et al. 2003; Klopocki et al. 2012). The minimal overlapping region of pathogenic SHFM3 duplications includes *BTRC*, *POLL,* and *DPCD* (Holder-Espinasse et al. 2019). The inversion described here is copy number neutral, suggesting that positional effects rather than gene dosage might be responsible for the phenotype. It includes a topologically associating domain boundary (Holder-Espinasse et al. 2019) and is likely to change the enhancer landscape at the SHFM3 locus leading to *FGF8* misregulation causing ectrodactyly.

**Fig. 2:**
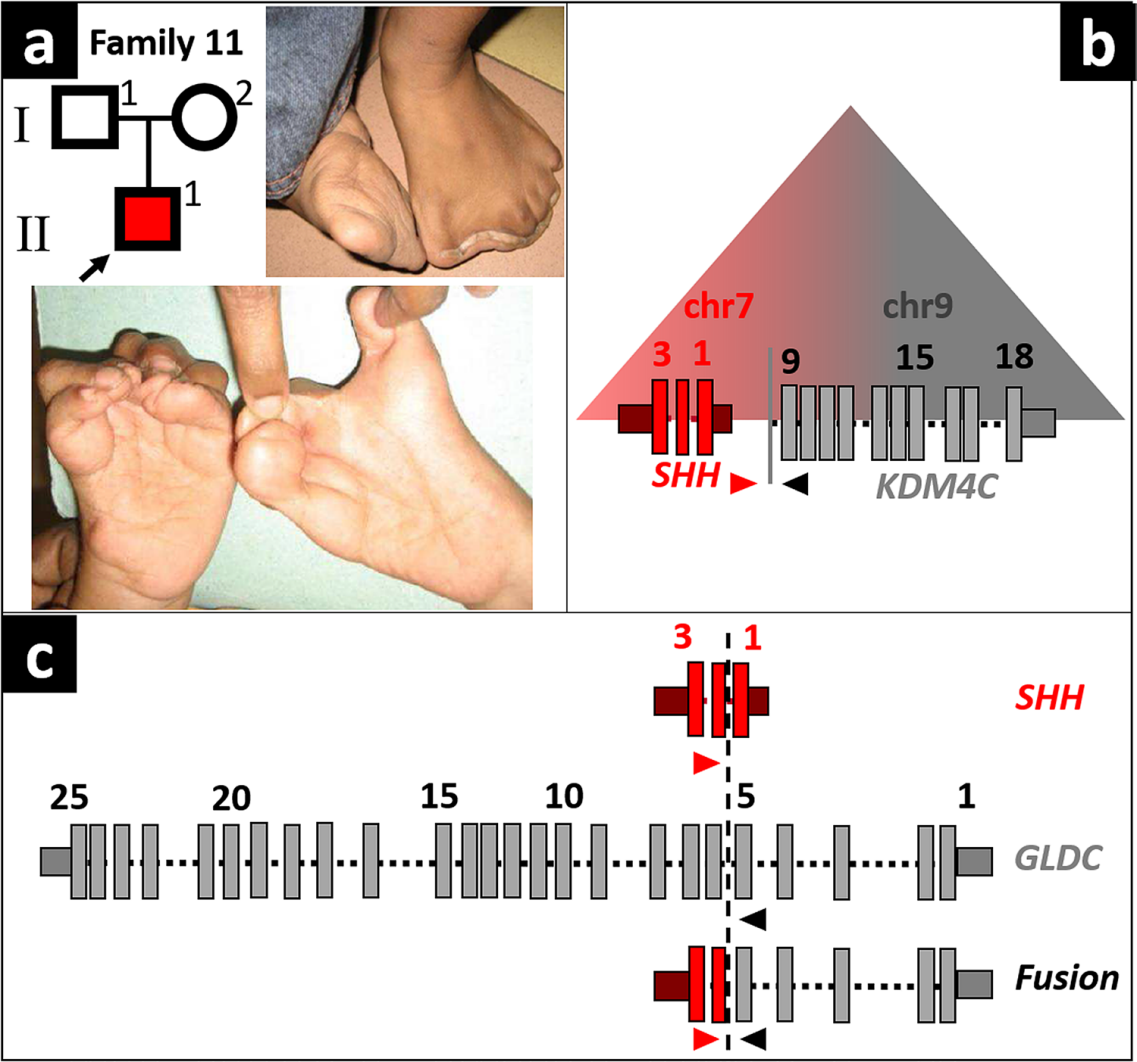
**a** Pedigree and phenotype of individual I11. **b** Potential neo-TAD at the fusion site. **c** Breakpoint and fusion sites between regions from chr7 and chr9

In individual I11, featuring bilateral mirror-image polydactyly of the hands and feet (Fig. 2a), CMA had detected a 300 kb amplification on chr7q36.1. We initially classified the variant as a variant of unknown significance, because the individual’s phenotype did not match that of an individual with muscular hypertrophy, reported to have a similarly sized and positioned duplication (Kroeldrup et al. 2012).

The amplification was identified again using GS. However, sequencing revealed that it was part of a complex structural variant containing two overlapping duplications (dup1 and dup2) at chr7q36.1 (Supplementary Fig. 7). The smaller dup2 shares the central breakpoint with dup1. The distal breakpoint of dup2 is positioned within dup1 in intron 1 of *SHH*, at chr7:155,603,964. GS data showed that the duplicons were not positioned *in tandem*, but are both fused to sequences originating from chr9p24.1. The distal breakpoint of dup2 was fused to intron 5 of *GLDC* and the distal breakpoint of dup1 to intron 8 of *KDM4C* (Fig. 2c; Supplementary Fig. 7). Analysis of the parents by Sanger sequencing showed that the structural variant occurred de novo in individual I11.

The mirror-image polydactyly of individual I11 shows striking phenotypic overlap with Laurin–Sandrow syndrome, which is caused by duplications of the *SHH* regulator ZRS, positioned in intron 5 of *LMBR1* on chr7p36.3, resulting in ectopic expression of *SHH* in the embryonic limb (Lohan et al. 2014). Both duplications do not include the ZRS and duplications of *SHH* itself have not been described to cause Laurin–Sandrow syndrome. However, a duplicated fragment containing *SHH* that is inserted into another domain, as observed in the de novo SV of I11, makes an ectopic expression of *SHH* in the embryonic limb very likely. We assume that the formation of an *SHH*-*KDM4C* neo-TAD, resulting in the misregulation of *SHH* by *KDM4C*-enhancers in the limb mesenchyme (note the known expression of KDM4C in embryonic vertebrate limb buds) is the most likely explanation for such an ectopic SHH-expression (Fig. 2b).

Therefore, we re-classified the complex SV involving *SHH* in individual I11 with bilateral mirror-image polydactyly as causative.

### Establishing *UBA2* as a novel disease gene

**Fig. 3:**
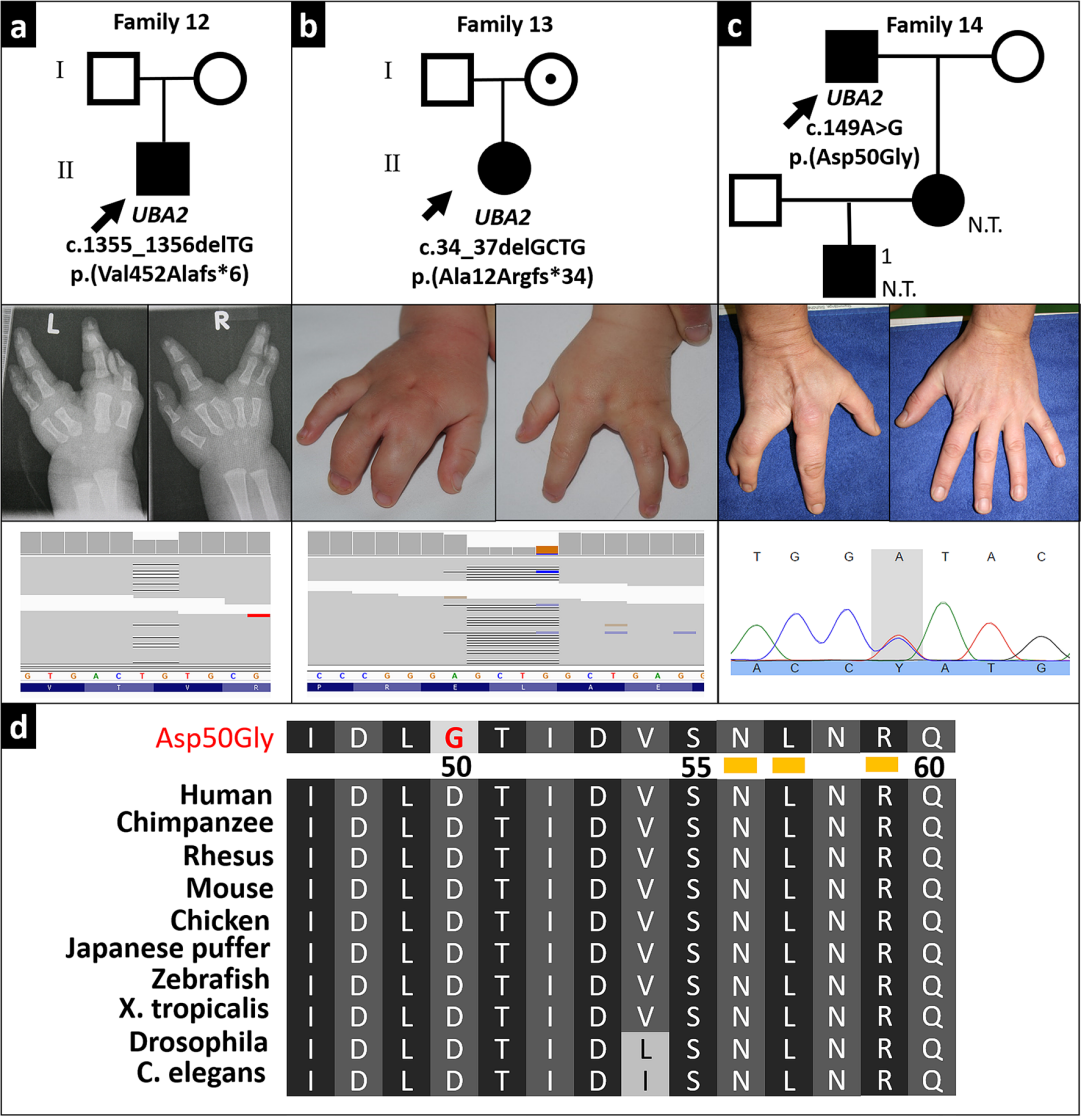
*UBA2* variants and ectrodactyly. **a**–**c** Patients with likely pathogenic UBA2 variants *upper panels*: pedigrees, N.T.: not tested; *middle panels*: characteristic limb malformations, *lower panels*: sequencing data. **d** conservation of Asp50 mutated in individual I14, numbers indicate amino acid residues, *yellow bars* highlight positions tested by Olsen et al. to cause loss of function when substituted by alanine (Olsen et al. 2010)

We also identified variants in new candidate genes. Two unrelated individuals with isolated split hand malformation featured different heterozygous frameshift variants in the *ubiquitin-like modifier-activating enzyme 2* (*UBA2*) (Fig. 3a, b). Individual I12 harboured the de novo variant NM_005499.3(*UBA2)*:c.1355_1356delTG;p.(Val452Alafs*6). Individual I13 inherited the variant NM_005499.3(*UBA2*):c.34_37delGCTG;p.(Ala12Argfs*34) from his apparently unaffected mother (no radiographs of her hands were available).

We classified the pathogenicity of these variants according to the ACMG guidelines. Both are null variants of *UBA2* which has a pLI-score of 1 (PVS1). c.1355_1356delTG occurred de novo in an individual with a negative family history (PS2). The variants are absent from the 1000 Genomes Project and the Exome Aggregation Consortium databases (PM2) and were predicted to be pathogenic by MutationTaster (PP3). *UBA2* variants have recently been described in individuals with ectrodactyly (Chowdhury et al. 2014; Abe et al. 2018; Yamoto et al. 2019; Aerden et al. 2020). Hence, we regarded UBA2 as a disease-associated gene and these variants as pathogenic (1PVS(+ 1PS) + 1PM + 1PP).

These findings prompted subsequent Sanger sequencing of *UBA2* in 24 unrelated families with ectrodactyly, who have been tested negative for variants in the established SHFM loci/genes. In one individual (I14) with unilateral split-hand malformation (Fig. 3c), we identified the missense variant NM_005499.3(*UBA2*):c.149A > G;p (Asp50Gly). The daughter of I14 and her son also feature ectrodactyly (PP4), but were unavailable for testing. Asp50 is part of a consecutive 15 amino acid sequence (Ile47 to Phe61) shared amongst all nephrozoan *UBA2* orthologues (Fig. 3d). Olsen et al. showed that variants of residues (Asn56Ala, Leu57Ala, Arg59Ala) of this element result in loss of UBA2 function, and found that the very residue mutated in individual I14, Asp50, forms hydrogen bonds with Asn177 and Thr178 essential for proper UBA2 folding and thus its function (PS3) (Olsen et al. 2010). The variant was also absent in the databases (PM2) and predicted to be pathogenic by MutationTaster (PP3). Hence, we classified these variants as likely pathogenic according to the ACMG’s guidelines (1PS + 1PM + 2PP).

### Novel candidate genes

Our analysis also revealed several novel, high-confidence candidate genes associated with limb defects. In individual I15 featuring severe mirror image foot polydactyly, we found a de novo frameshift variant in the gene encoding the high mobility group box 1 protein (HMGB1) (Supplementary Fig. 8). NM_002128.7(*HMGB1*):c.551_554delAGAA;p.(Lys184Argfs*44) leads to the replacement of the protein’s entire C-terminal 30-residue acidic tail by 41 other unrelated residues. The tail is normally formed by an Asp/Glu-repeat element, which is highly conserved among *HMGB1* orthologues. This repeat element stabilizes HMGB1’s secondary structure and is crucial for its DNA-bending capacity (Belgrano et al. 2013; Anggayasti et al. 2020). The variant is not only absent from the databases but also no variant listed in gnomAD contains an amino acid residue except Glu or Asp in the acidic tail domain. *HMGB1* has a pLI score of 1. In mouse and zebrafish studies, HMGB1 has been shown to regulate digit number during embryonic limb development by interacting with WNT, BMP, and SHH (Itou et al. 2011). We, therefore, consider *HMGB1* to be a novel candidate gene for mirror image foot polydactyly.

Individual I16, who featured short stature, absent distal phalanges of the 5th fingers and toes, and dysplastic middle phalanges of the toes carried a de novo missense variant in the gene encoding semaphorin 3D (*SEMA3D*) (Supplementary Fig. 9). NM_152754.3(*SEMA3D*):c.1918G > A;p.(Asp640Asn) is absent from the 1000 Genomes Project database and is listed only 4 times in the Genome Aggregation Database (gnomAD). The Asp640 residue in the immunoglobulin-like domain of *SEMA3D* is highly conserved amongst vertebrates. The variant is predicted to be pathogenic by MutationTaster. SEMA3D regulates neural crest cell differentiation and is involved in the organogenesis of the heart (Sanchez-Castro et al. 2015), parathyroid gland (Singh et al. 2019), and, notably, limbs (Govindan et al. 2016). We, therefore, consider it a candidate gene for short stature with limb abnormalities.

In individual I17 we identified a paternally inherited frameshift variant in the *aldehyde dehydrogenase 1 family member A2* gene (*ALDH1A2*) encoding retinaldehyde dehydrogenase 2 (Supplementary Fig. 10). Both, the patient and her father feature isolated cutaneous syndactyly of the fingers III–IV and the toes II–III. The variant NM_003888.4(*ALDH1A2*):c.35delT;p.(Val12Glyfs*31) is absent from the databases and is predicted to be disease-causing by MutationTaster. ALDH1A2 is a direct target of HOXA13 and plays a key role in vertebrate digit development by regulating, in particular, interdigital programmed cell death (Shou et al. 2013). Rescued *ALDH1A2* knockout mice show reduced interdigital cell death and thus impaired digit separation during limb development resulting in syndactyly (Zhao et al. 2010). It is, therefore, likely that *ALDH1A2*:c.35delT caused the phenotype of syndactyly in individual I17 and her father.

### Identification of noncoding variants

So far, the interpretation of disease-related variation has been focused on protein-coding DNA and the identification of variants that directly result in the disruption of specific gene functions.

Here, we aimed to develop a framework to prioritize a large number of noncoding variants from GS studies, by combining mouse genetic and human functional epigenetic data with in vivo-validated enhancer sequences. We then defined a limb-specific regulome that we used to filter all noncoding variants (Materials and Methods). Our potentially disease-relevant limb-specific regulome consists of 5,591,007 sites covering in total 7,294,220 bp, i.e. 0.24% of the human genome (hg19).

**Fig. 4:**
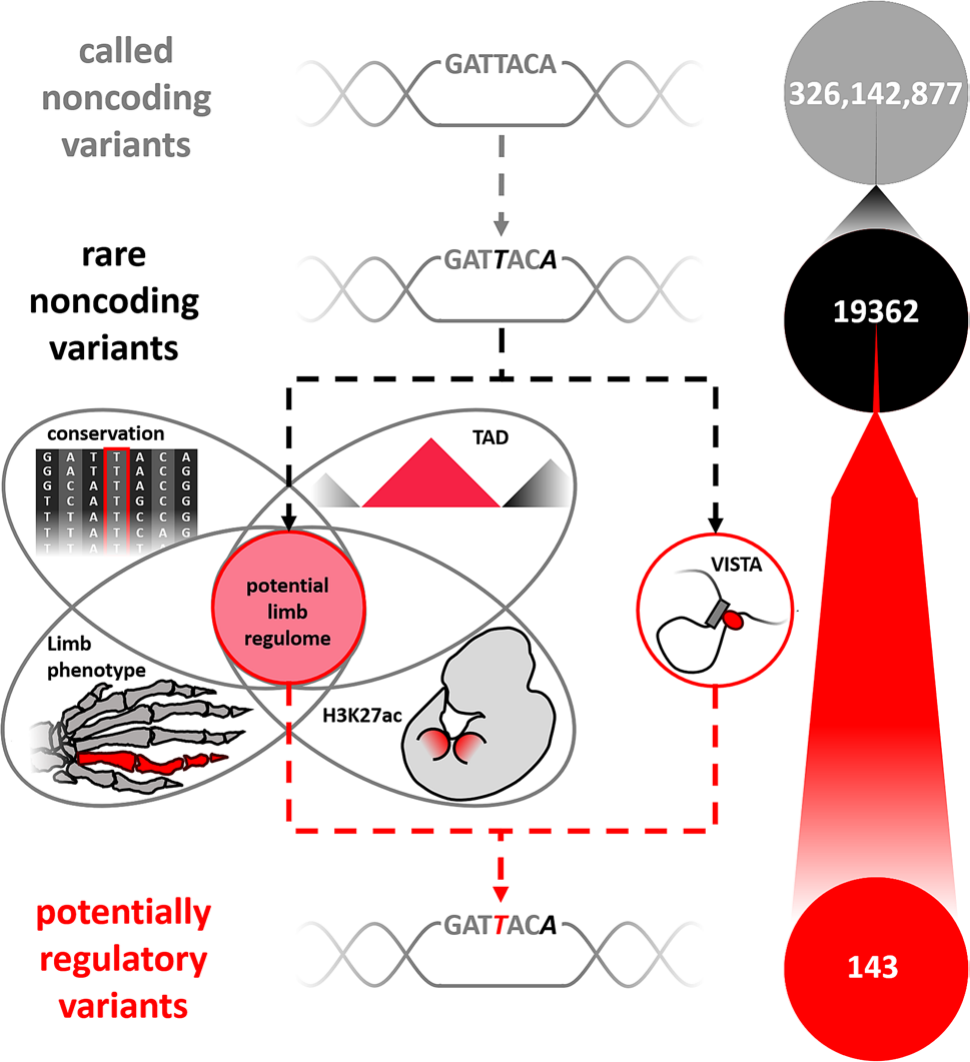
Pipeline of noncoding data analysis

Overall, we identified 19,362 rare noncoding SNVs in the 69 index patients, of which 143 were located within the limb regulome (Fig. 4). First, we focused on the de novo variants and identified 6 calls located in potential regulatory elements. Two variants were excluded because they were called in cases with (likely) pathogenic coding or structural variants.

Individual I18 presenting with bilateral syndactyly of fingers II-V featured the de novo call chr1:41948304AAG > A in intron 2 of *EDN2*. The position shows increased acetylation of H3K27 in human limb buds. The 2 bp deletion also removes one element of a 6-AG-repeat whose length is not conserved in vertebrates. Furthermore, *EDN2* encodes endothelin 2, a potent vasoconstrictor with no evident link to limb development.

Individual I19 showing upper limb amelia featured three calls (chr5:157285900CACGTGGG > C, chr5:157285909CTCGG > C, chr5:157285915CACAACTG > C) referring to the same indel in intron 1 (15 bp downstream of the first exon–intron boundary) of *CLINT1.* However, *CLINT1* shows only a moderate pLI score (0.54) and there is no evidence other than increased H3K27ac marks of its promoter region in human limb buds linking it to limb development.

We were not able to identify any rare variants in validated VISTA enhancers that showed enhancer activity in the limb bud.

Next, we focused on noncoding variants that were located close to one another in more than one case. In total, 3425 rare noncoding variants in the unsolved cases were positioned 300 bp or less apart from a variant in another unsolved case. 16 of these calls were located within the limb regulome, but in five of these variants, the other variant was positioned outside of the limb regulome.

In two cases both variants were positioned within the limb regulome and within 300 bp: individual I21 and individual I22, both showing finger syndactyly, harbored the overlapping deletions chr22:24552064GGGGGCCGGGACTGGGGCCGGGACT > G and chr22:24552086ACTGGGGCCGGGG > A, respectively. The deletions are positioned in intron 29 of *CABIN1*, in an evolutionarily partially conserved element, that shows increased H3K27ac marks in human embryonic limb buds. However, both deletions were inherited from unaffected parents.

Eight of the close variants were double hits (i.e. we detected rare calls not in just one but two index patients at four positions of the potential limb regulome). However, none of these four pairs of index patients showed overlapping phenotypes.

We identified no coding variant of a known limb disease gene in trans with a conserved, rare noncoding variant of the same TAD.

In summary, despite extensive efforts, we were not able to identify any noncoding SNVs that showed convincing evidence to be causal in congenital limb malformations.

## Discussion

In this study, we set out to determine the potential of GS as a comprehensive diagnostic tool to determine all kinds of genetic variants associated with congenital limb malformation.

In our cohort of patients with congenital limb malformations, GS was able to detect both previously described and novel causative genetic variants in already established limb malformation associated genes. In addition, it enabled the identification of three candidate genes and the independent verification of the novel disease gene *UBA2* for causing ectrodactyly (Yamoto et al. 2019). Our approach was able to detect SNVs and structural variants. Finally, GS proved to be a powerful strategy to identify genomic variants previously missed by most other approaches, including repeat expansions and complex structural variants. In total, we identified variants that we consider to be likely pathogenic/disease-associated in 12 of 69 cases (17.4%). This diagnostic yield is comparable to the recent landmark study conducted by the British National Health Service that used GS in cohorts with other congenital disease entities (Turro et al. 2020). A clear advantage of GS compared to most other technologies is the ability to detect copy number neutral variants and to gain position information on CNVs. In our cohort of only 69, we were able to detect two complex variants, an inversion at the *FGF8* locus and a translocated triplication including the *SHH* gene. Both were missed by standard technologies. Further research is necessary to clarify their exact pathomechanisms. The variants identified in the genes *HMGB1*, *SEMA3D* and *ALDH1A2* are all likely to cause loss of function. The genes were previously associated with vertebrate limb development in animal studies and the variants either arose de novo or segregate with the respective phenotype. However, we could not identify unrelated individuals featuring comparable variants in these candidate genes and similar phenotypes. Future research is necessary to identify such to establish the described candidates as disease genes. Our findings once again highlight the role of GS as an attractive one-test-for-all strategy for clinically very heterogeneous cohorts such as congenital malformation syndromes or intellectual disability (Gilissen et al. 2014; Turro et al. 2020). The total cost of the various conventional tests currently used in clinical routine far exceeds that of trio GS.

One of the main challenges of GS data is the medical interpretation of changes in the noncoding DNA. While most clinical GS studies tend to ignore noncoding SNVs (Gilissen et al. 2014) there are recent anecdotal reports of noncoding variants as the cause of Mendelian disorders (Lettice et al. 2003; Jeong et al. 2008; Albers et al. 2012; Bhatia et al. 2013; Weedon et al. 2014; Bae et al. 2014), although there is no established systematic approach, yet. Therefore, we set out to develop a framework to prioritize such noncoding variants associated with congenital limb malformation. We used a combinatorial approach of mouse and human epigenetic data, in vivo validated enhancer sequences, knock-out mice, and the recent knowledge about 3D genome folding, and the cis-regulatory architecture of the genome to define a limb regulome. This limb regulome consists of 0.24% of the genome and includes all known in vivo-validated limb enhancer elements. Contrary to our expectation, we could only identify candidate loci, but no definitely pathogenic noncoding variants. These findings are in stark contrast to our recent study where we demonstrated that CNVs affecting noncoding regulatory elements are a major cause of congenital limb malformations (Flöttmann et al. 2018).

While our results suggest that GS is sensitive to classical sequence variants, it is noteworthy that the method cannot detect epigenetic variants. Epimutations (e.g. imprinting defects) are known to cause inheritable human disease. However, to our knowledge, no epimutation has been linked to congenital limb malformation yet.

Tools for the analysis of GS data are continuously being developed further and the precision of algorithms to call structural variants can certainly be improved. We expect the diagnostic rate to increase steadily with the accuracy of the instruments invoked to analyze GS data.

## Supplementary Information

Below is the link to the electronic supplementary material.

Supplementary file1 (XLSX 20 KB)

Supplementary file2 (XLSX 9 KB)

Supplementary file3 (XLSX 9 KB)

Supplementary file4 (PNG 213 KB)

Supplementary file5 (JPG 789 KB)

Supplementary file6 (JPG 736 KB)

Supplementary file7 (PNG 685 KB)

Supplementary file8 (PNG 587 KB)

Supplementary file9 (JPG 1173 KB)

Supplementary file10 (PNG 512 KB)

Supplementary file11 (PNG 94 KB)

Supplementary file12 (PNG 412 KB)

Supplementary file13 (PNG 1071 KB)

## Data Availability

Code and data (pseudonymized, grouped where possible, and minimized) are available upon reasonable request.
