# Supplementary figures and images for: Genome sequencing in families with congenital limb malformations
Source: Hum Genet. 2021 Jun 22;140(8):1229–39. doi: 10.1007/s00439-021-02295-y (PMC8263393; doi:10.1007/s00439-021-02295-y)

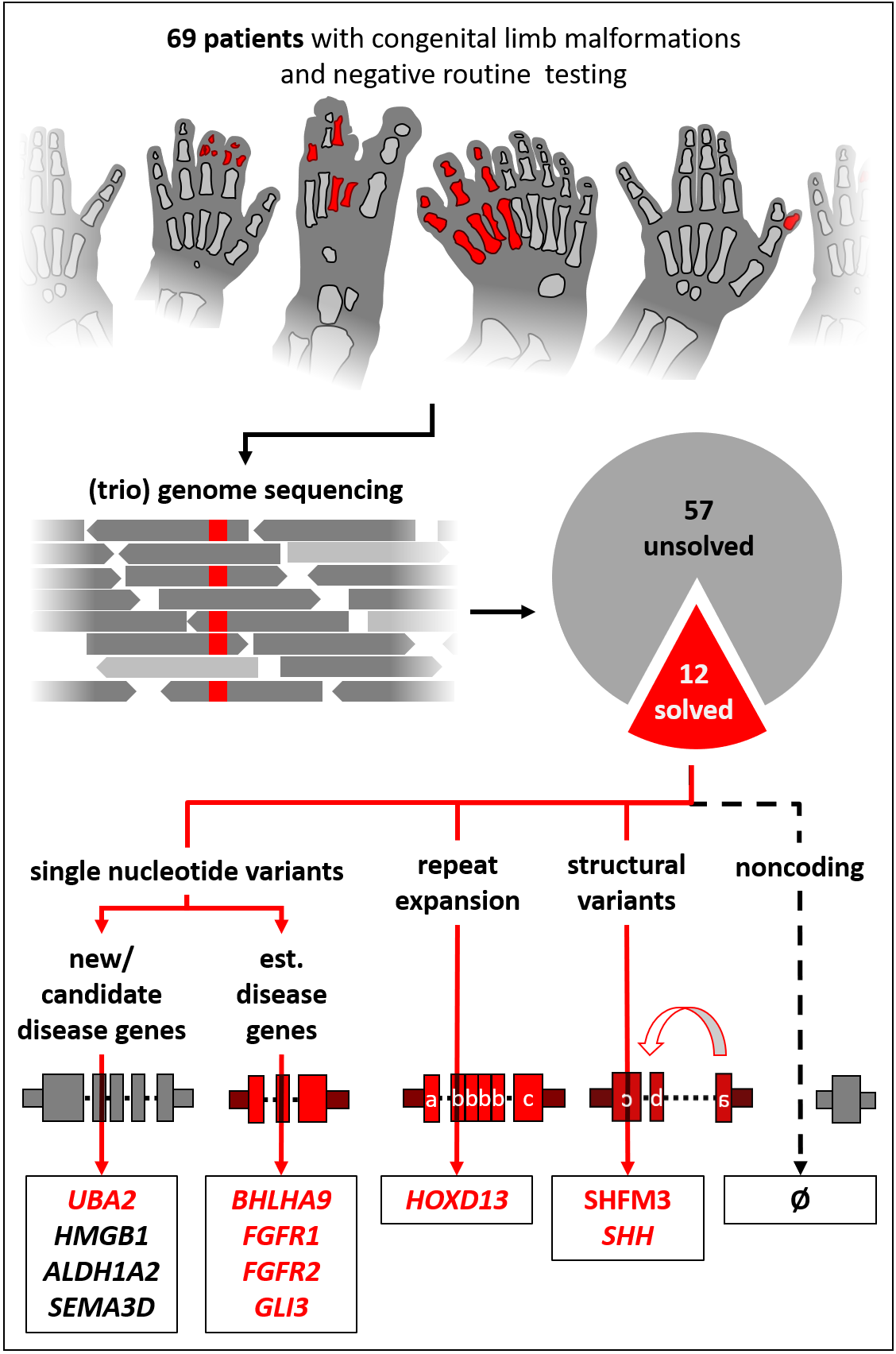

Supplement: Supplementary file 4 — Supplementary file4 (PNG 213 KB) [file 439_2021_2295_MOESM4_ESM.png]

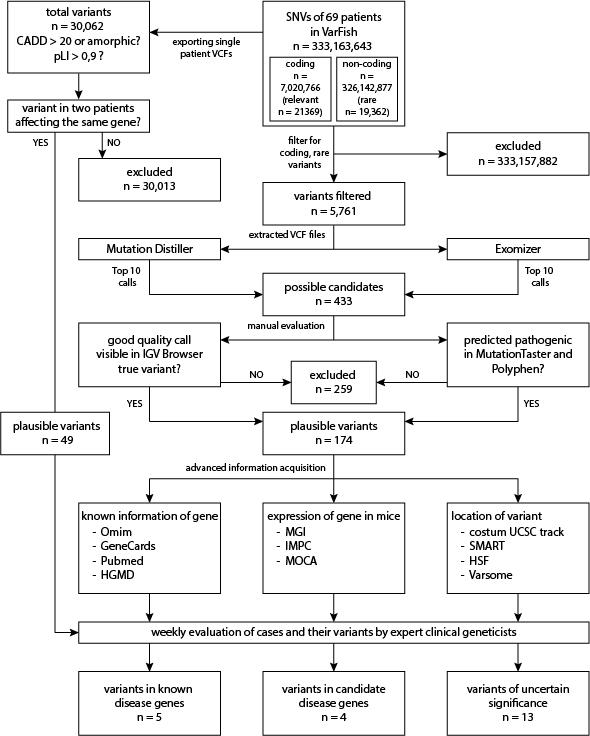

Supplement: Supplementary file 5 — Supplementary file5 (JPG 789 KB) [file 439_2021_2295_MOESM5_ESM.jpg]

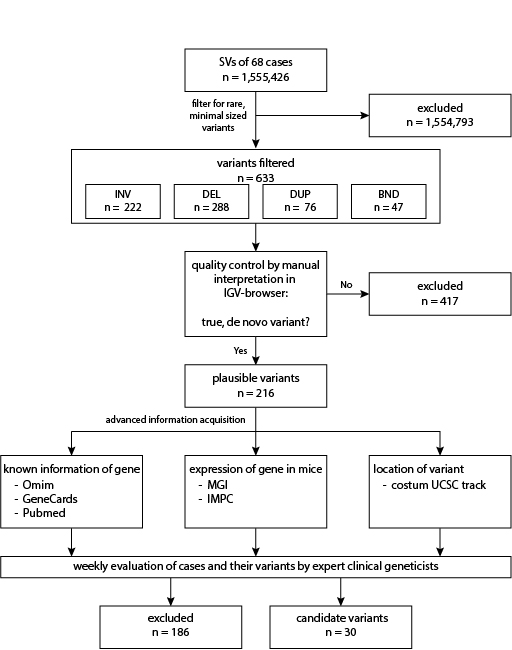

Supplement: Supplementary file 6 — Supplementary file6 (JPG 736 KB) [file 439_2021_2295_MOESM6_ESM.jpg]

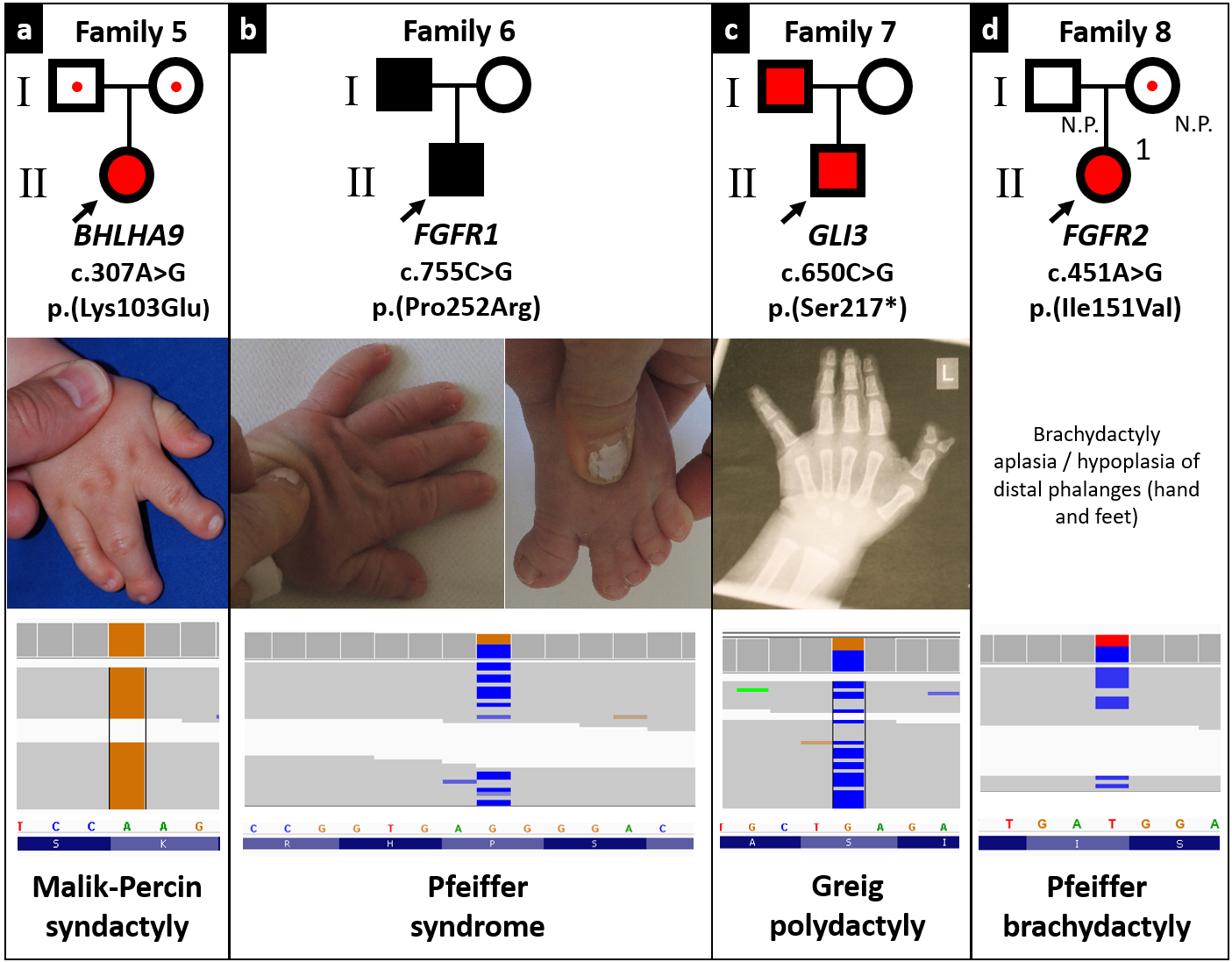

Supplement: Supplementary file 7 — Supplementary file7 (PNG 685 KB) [file 439_2021_2295_MOESM7_ESM.png]

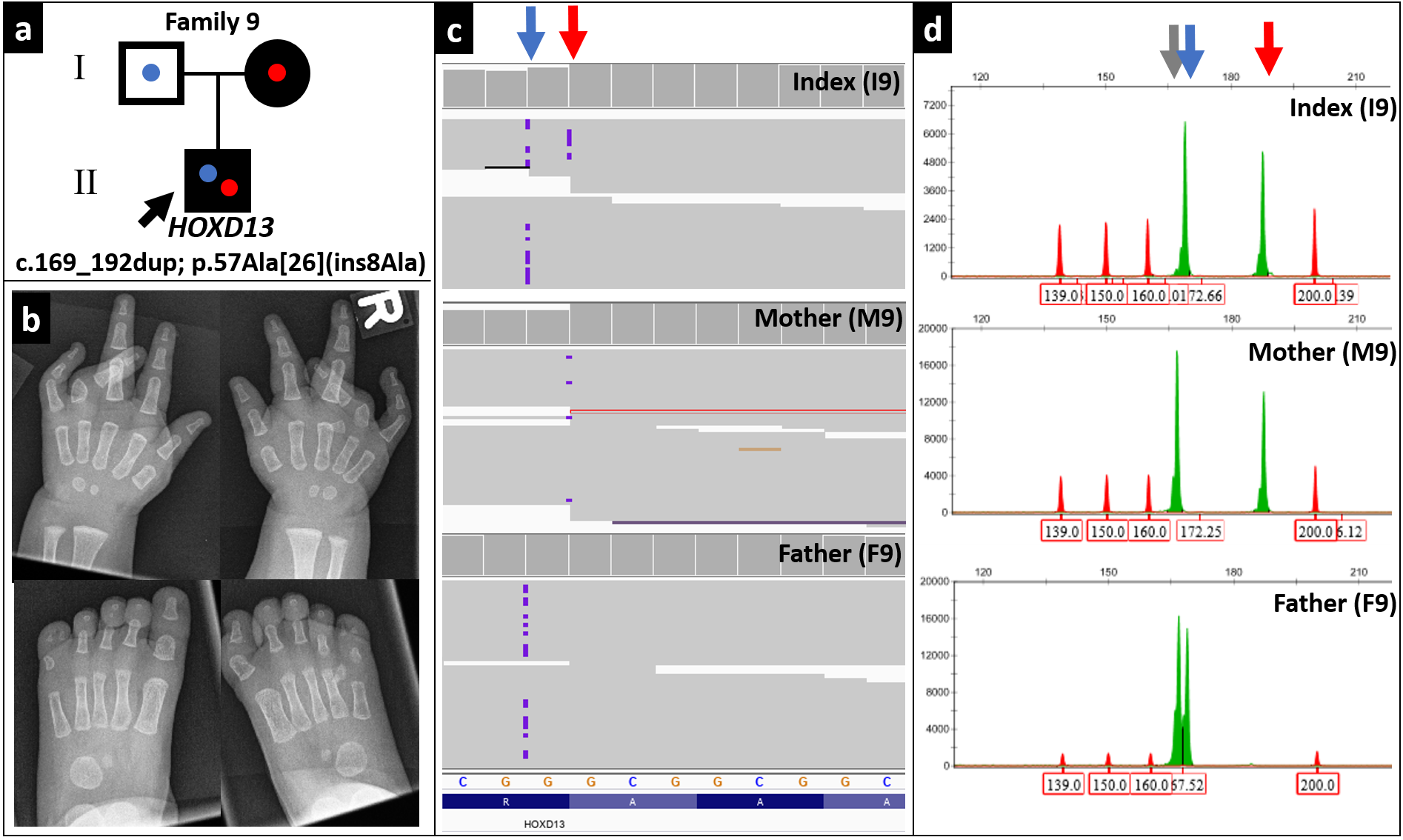

Supplement: Supplementary file 8 — Supplementary file8 (PNG 587 KB) [file 439_2021_2295_MOESM8_ESM.png]

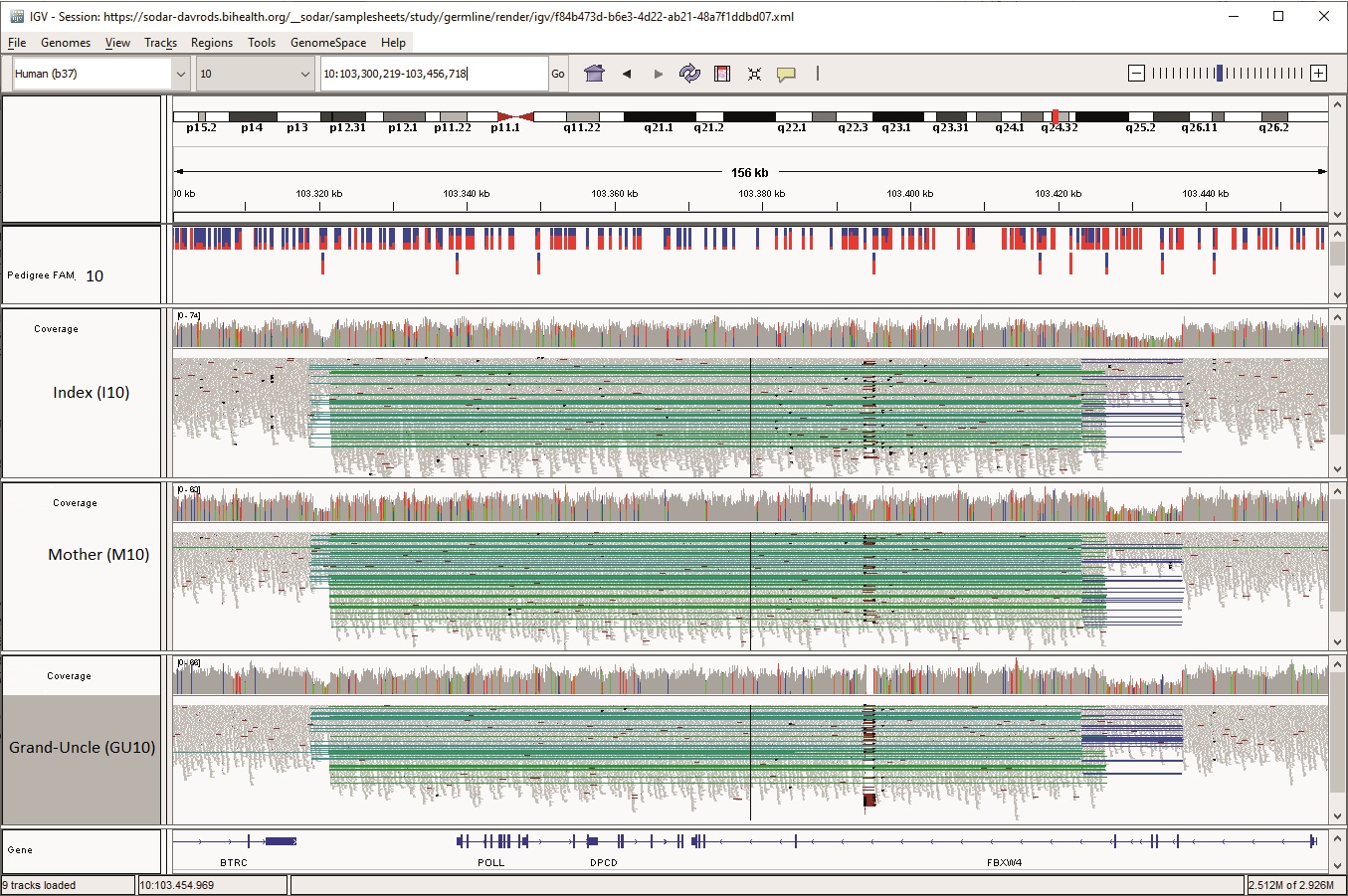

Supplement: Supplementary file 9 — Supplementary file9 (JPG 1173 KB) [file 439_2021_2295_MOESM9_ESM.jpg]

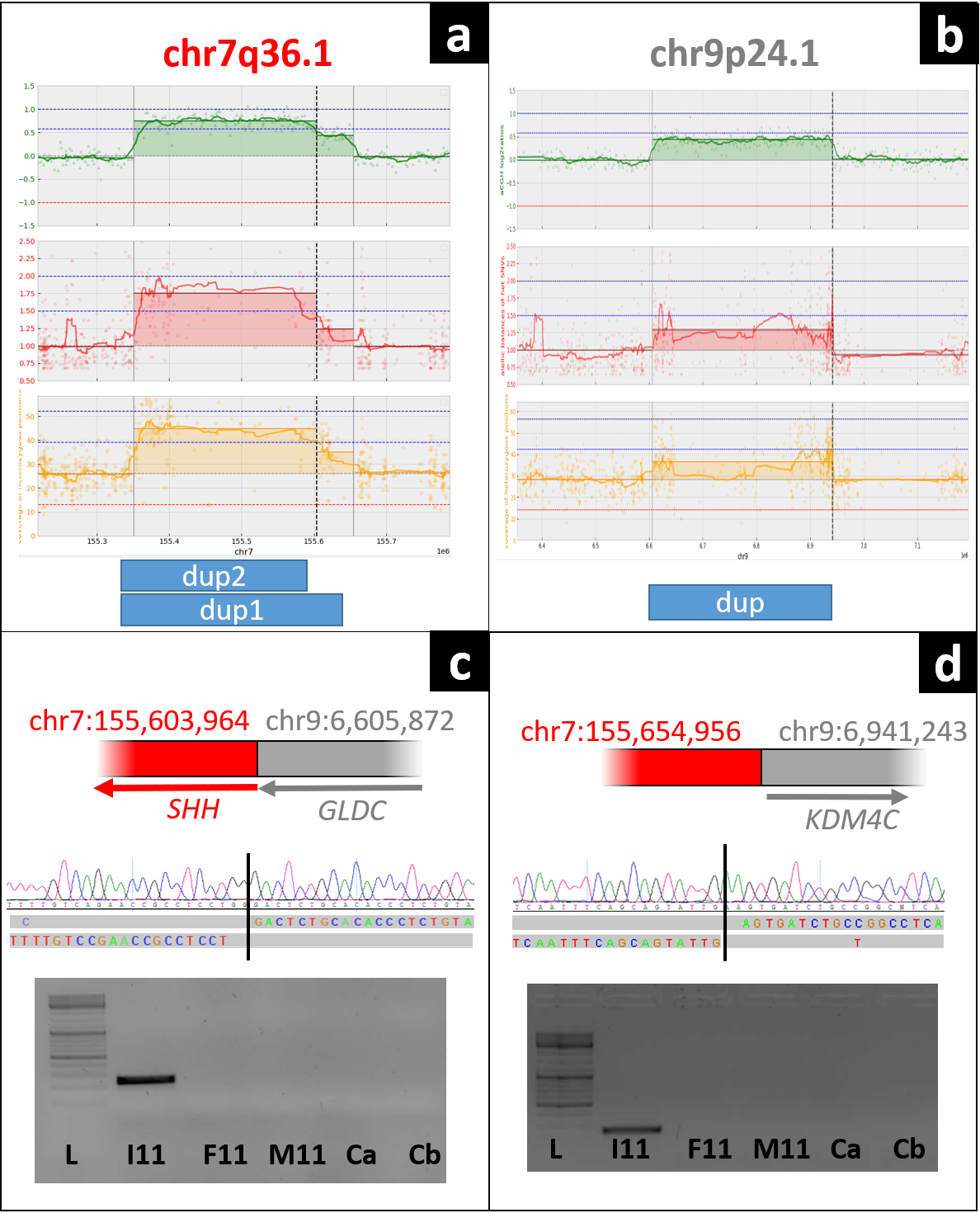

Supplement: Supplementary file 10 — Supplementary file10 (PNG 512 KB) [file 439_2021_2295_MOESM10_ESM.png]

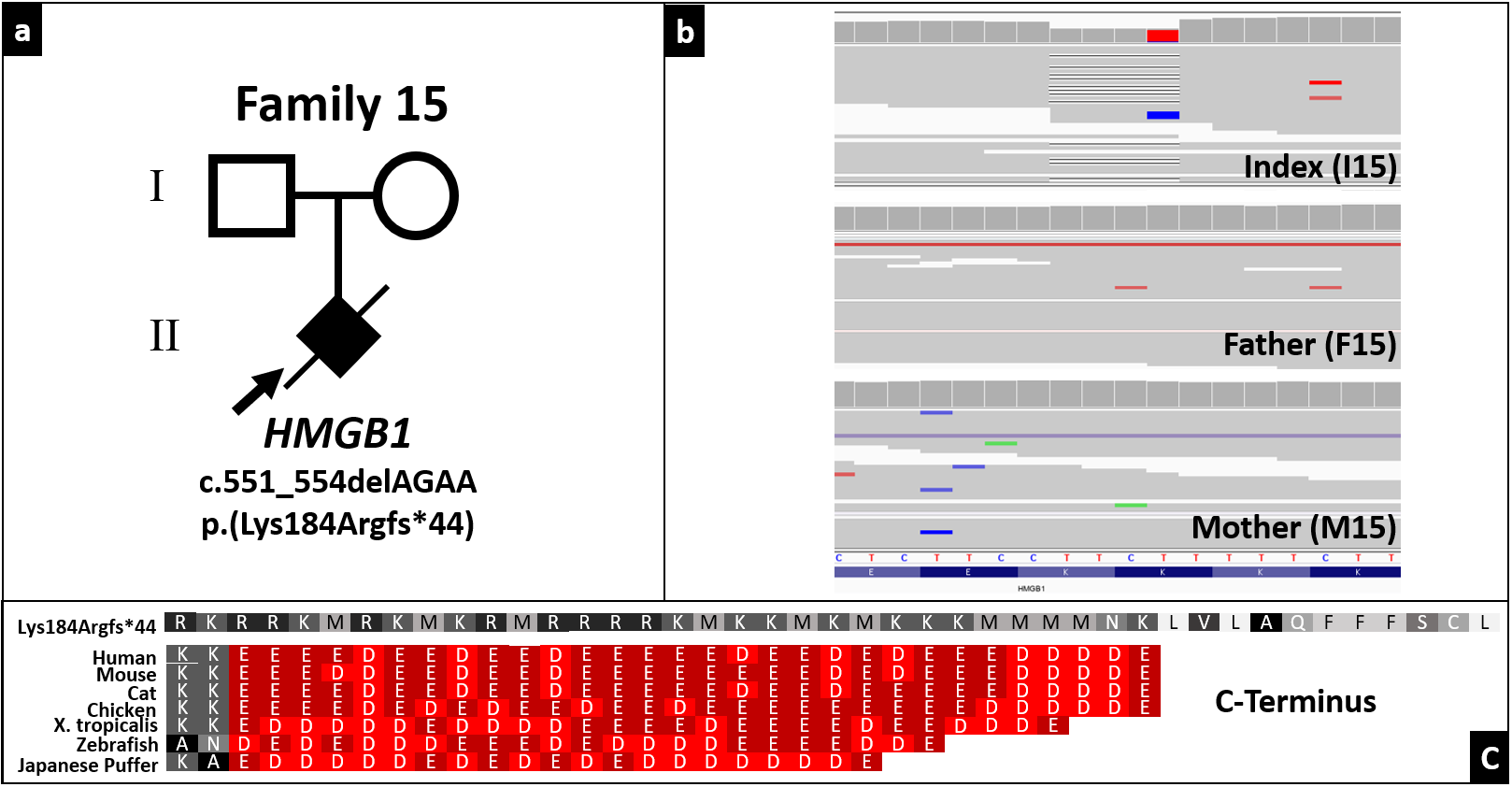

Supplement: Supplementary file 11 — Supplementary file11 (PNG 94 KB) [file 439_2021_2295_MOESM11_ESM.png]

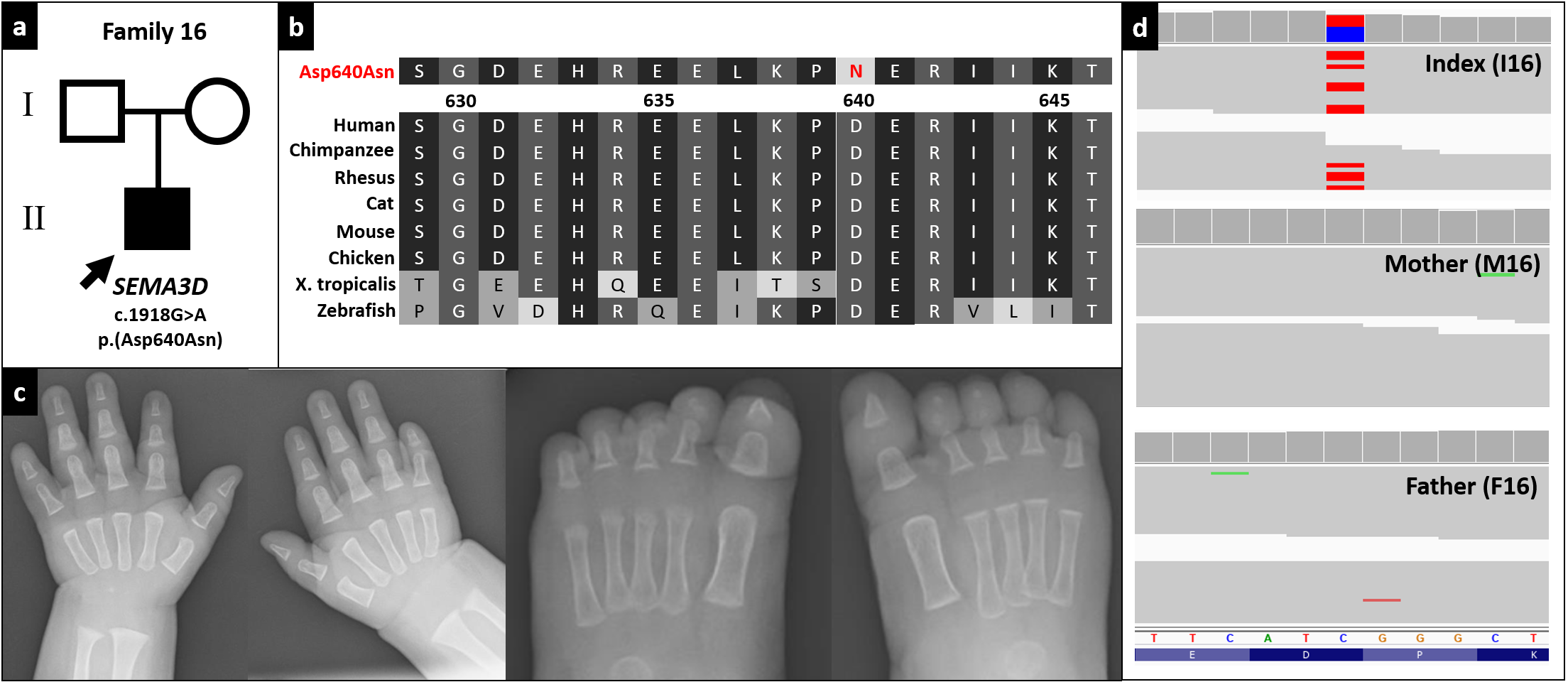

Supplement: Supplementary file 12 — Supplementary file12 (PNG 412 KB) [file 439_2021_2295_MOESM12_ESM.png]

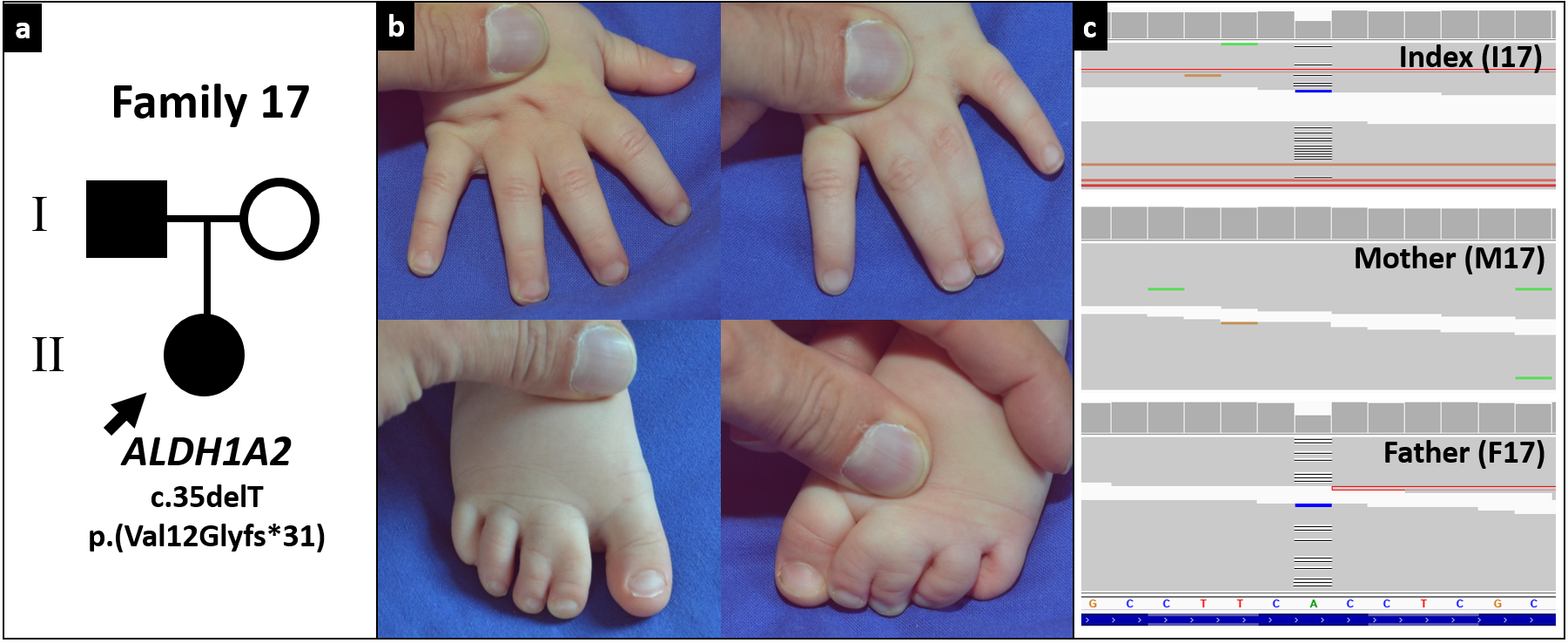

Supplement: Supplementary file 13 — Supplementary file13 (PNG 1071 KB) [file 439_2021_2295_MOESM13_ESM.png]
